# Tropical Hæmatology

**Published:** 1910-01-22

**Authors:** George C. Low

**Affiliations:** Lecturer in Tropical Diseases to the Post Graduate College, West London Hospital, etc.


### PATHOLOGY.

### TROPICAL HÆMATOLOGY.

### Romanowsky's Chromatin Stain and its Modifications.

By GEORGE C. LOW, M.B.; Lecturer in Tropical Diseases to the Post Graduate College, West London Hospital, etc.

Since the introduction of Romanowsky's chromatin stain, great advances have been made in the study of special blood parasites. Without such a stain or its modifications many of the parasites—trypanosomes, Leishman bodies, etc.—cannot be seen. The staining procedure and preparation of the stain, as first invented, were long and tedious, and more practical modifications, of which Leishman's was the first important one, quickly followed. Many such now exist, and in certain cases, for staining special parasites, one modification has advantages over the others. As many of the chief modifications are of French or German origin and insufficiently known, it may be useful to describe them in detail here.

#### Original Method.

The original stain had as its constituents: Medicinal methylene blue (Grubler's), eosin extra B.A. or A.G., and sodium carbonate pure, the method of making it up being as follows. Two stock solutions are taken.

| | | |
|---|---|---|
| Sol. A.— | Methylene blue ... ... | 1 gram. |
| | Sod. carb. ... ... ... | .5 gram. |
| | Water ... ... ... | 100 c.c. |

This is placed in a hot incubator for two or three days. It should then become purple, *e.g.* polychrome, so that when combined with eosin, a third stain which picks out the chromatin should be produced.

| | | |
|---|---|---|
| Sol. B.— | Eosin ... ... ... | 1 gram. |
| | Water ... ... ... | 1000 c.c. |

For staining: dilute stock solutions 1 in 20 with water.

*To use*: Equal portions (about 4 c.c. of each) are poured into a porcelain dish and mixed by shaking. The cover glasses or slides are placed in this and left for ten minutes to half an hour or longer. They are then washed well with water, dried in the air, and mounted. If too blue they are washed longer in water, or rapidly in spirit and water.

#### Modifications.

1. *Leishman's modification*: (*B.M.J.*, Sept. 21, 1901.)

Sol. A.—Medicinal methylene blue 1 per cent. in distilled water; .5 per cent. sod. carbonate.

This is heated to 65° C. for twelve hours, allowed to stand for 10 days afterwards, and at the end of that time the blue will be found to be polychrome.

Sol. B.—Eosin extra B.A. (Grubler) 1 in 1000 solution in distilled water.

Equal quantities of A and B are mixed in an open vessel and stirred. After stirring allow to stand for 6 to 12 hours. The precipitate that then appears is collected by filtering through filter paper, washed with distilled water until washings are colourless or only pale blue, dried and powdered, the residue being kept stored in glass phials.

To make up the stain take of the dry powder .15 gram, dissolving this in 100 c.c. of pure methylic alcohol (Merck's for analysis). This forms the staining solution.

#### Method of Staining.

*To Stain*: No preliminary fixing is required. Pour on stain on cover glass or slide; leave for ½ to 1 minute. Next add double the quantity of distilled water, mix, allowing this to remain on for 5 minutes. Next wash in distilled water and leave a drop of this on for 1 minute. Dry in the air and mount. Rain water or soft tap water may be used if no distilled water is available. Different samples of the powder made as above vary considerably in their staining power, but now Burroughs and Wellcome have put the stain up in tabloid form, samples being very constant in their action and saving all the original trouble of making the powder. Full directions are given for use with each tube of tabloids; these are very simple. One tabloid is to be dissolved in 10 c.c. pure methylic alcohol. It is useful to have two little drop bottles holding 60 c.c. each, say. Into one drop 5 tabloids, then 50 c.c. pure methylic alcohol. Into the other pour distilled water. Use as above. No fixing; drop on stain, leaving for 1 minute, equal parts of distilled water 5 minutes, distilled water alone 1 minute. Dry in the air or by blotting, and either mount in Canada balsam or keep unmounted (latter do not fade so quickly). Staining by this method will do very well for all tropical blood parasites, and as it is so simple and easy to use it is the one in general use in this country.

*Other Methods of Rendering Methylene Blue Polychrome.*—The following methods may also be employed for rendering methylene blue polychrome:—

(*i.*) Heat medicinal methylene blue solution in steam steriliser two hours on three consecutive days.

(*ii.*) Wright's method. Take sodium bicarbonate ½ gram, med. methylene blue 1 gram, water 100 c.c. Steam this for one hour.

(*iii.*) Silver method. Take fresh oxide of silver. This is got by adding silver nitrate ($AgNO_3$) to a solution of sodium hydrate. Wash the precipitate that results (silver oxide) till neutral to litmus paper; then add it to a saturated solution of medicinal methylene blue. Let stand for 24 hours. Decant off from the silver, and filter. The methylene blue should now be polychrome.

2. *Nocard's Method of Staining* (as communicated to author by Dr. Brumpt, of Paris).—This method is said to be specially good for staining piroplasmata, and it is also good for trypanosomes. The stain does not seem to fade so quickly as in some of the other modifications, and this is an advantage, even though it takes longer to do.

Three solutions are used:

| | |
|---|---|
| Sol. A.—Chlorhydrate methylene azur (Grubler) ... ... | .5 gram. |
| Eau phénique (½ per cent. .5 gram) ... ... ... | 100 c.c. |
| Sol. B.—Extra B.A. eosin (De Max) | 1 gram. |
| Water ... ... ... | 4000 c.c. |

*To use*: Fix films in absolute alcohol. Take one drop of A and 12 drops of B, spreading over slide and leaving for half an hour. At the end of that time wash with ordinary water thoroughly and stain for 30 seconds with

Sol. C.—Tannin Orange (Grubler).

Wash, dry, and mount.

3. *Zieman's Blood Stain.*—Borax is used in this stain. Results are much more uncertain than with previous methods:

Sol. A.—Methylene blue (Höchst) ... 1 gram.

Dissolve this in 100 grams of warm distilled water in a flask, and then to this add 2.5 grams pure powdered borax. Allow solution to stand a day or two; shake.

| | |
|---|---|
| Sol. B.—Eosin (Höchst) (A.G. or B.A.) ... ... ... | 1 gram. |
| Water hot ... ... | 1000 c.c. |

*To use*: Fix films in absolute alcohol 10 minutes; dry; 3 parts of A are mixed with 12 parts of B. Pour some of this over preparation to cover it. A metallic film then appears; remove this after 5 minutes, or latest 10, with blotting paper. Rinse in water, then in acetic acid .1 in 1000 for a second or two, then in 60 to 80 per cent. alcohol for two or three seconds. Rinse in water, dry, and mount.

### Laveran and Mesnil's Method.

4. *Laveran and Mesnil's Method.*—This method is used by Laveran and Mesnil for staining trypanosomes in blood films or in cultures. The results are probably no better than by Leishman's or Nocard's stain, and the technique is also more complicated. What is called Borrel blue is employed. This is made by adding a small quantity of freshly precipitated silver oxide to a saturated watery solution of methylene blue (Höchst).

*To Stain*: 1. Make thin films, place as soon as dry in absolute alcohol for 5 minutes. 2. Transfer the film, without drying, to the stain, which is composed of—

| | |
|---|---|
| Borrel blue ... ... ... ... | 1 part. |
| 1 per cent. eosin (Höchst) ... ... | 4 parts. |
| Distilled water ... ... ... ... | 6 parts. |

The film must be placed in the stain the moment the latter is mixed—

3. After staining for 15 minutes wash well in water.

4. Place in 5 per cent. tannic acid solution for 10 minutes.

5. Wash in water, dry.

If a precipitate is deposited on the film, clear in clove oil, wash in xylol, mount; or remove by placing film or slide in water, and then brush lightly with a camel's hair brush.

### Giemsa's Method.

5. *Giemsa's Stain.*—This is the best stain for demonstrating the Spirochæta pallida (Schaudinn), but it may also be used for malaria and other parasites. The following reagents are used:

| | |
|---|---|
| 1. Azur II. rosin ... ... ... | 3,0 g. |
| 2. Azur II. ... ... ... ... ... | 0,8 g. |
| 3. Glycerin (Merck, Chemisch Rein) | 250,0 g. |
| 4. Methyl alkohol (Kahlbaum I.) ... | 250,0 g. |

One and two are mixed together, dried in the desiccator, then powdered, and dissolved in the 250 grams of glycerin at 60° C. The 250 grams of methyl alcohol, first warmed to 60° C., are then added, and the mixture is left standing for 24 hours. After this it is filtered and then is ready for use.

*To Use*: 1. Thin films of blood or syphilitic material dried in the air, are fixed in absolute alcohol for 15 to 20 minutes; dry by blotting with filter paper.

2. Dilute the staining solution with distilled water, shaking in a graduated reagent glass, in the proportion of 1 drop of stain to about 1 c.cm. of water.

3. Cover specimen with this and stain 10 to 15 minutes (previous heating to 40° C. is said to intensify the stain).

4. Wash in a stream of water.

5. Dry and mount.

The diluted stain can be conveniently kept in an ordinary drop-bottle, remembering, however, before one puts it in to wash the bottle out with absolute alcohol, and also to see that when not in use the stopper is turned, so as to exclude all air. A modified procedure to this for the spirochæta pallida (treponema pallidum) is as follows: Thin smears of material from syphilitic lesions are dried and fixed in absolute alcohol for 10 minutes. Stain 16 hours in a freshly made mixture of 12 parts Giemsa's eosin solution (2.5 c.c. of 1 per cent. eosin in 500 c.c. water), 3 parts Azur No. 1 (sol. 1 in 1,000 in water), and 3 parts Azur No. 2 (sol. 0.8 to 1,000 water); briefly rinse in water, dry, and mount in cedar-wood oil.

The advantage of the use of any of the modifications of Romanowsky's original stain is that chromatin is specially picked out and stained a reddish purple colour. A young malarial parasite will thus show up as a signet ring, the signet or nucleolus being reddish purple, the protoplasm, or ring proper, being blue, while the corpuscle in which the parasite is lying should stain a faint pink. Older parasites will show more blue (protoplasm) with disappearance of the vesicular nucleus and scattering of the chromatin through the body of the parasite. In Leishman bodies the two little chromatin particles appear very distinctly, as does also the nucleus and centrasome in the case of trypanosomes. Besides staining parasites, the different leucocytes are also well brought out in blood films, so that the stain is also useful for ordinary blood work; it has, as a matter of fact, to a very great extent replaced the old hæmatoxylin and eosin methods. It has also been employed for staining sections, but the technique for this is necessarily more difficult and is not so much within the province of the ordinary individual.

